# Commissioning, clinical implementation, and initial experience with a new brain tumor treatment package on a low‐field MR‐linac

**DOI:** 10.1002/acm2.13919

**Published:** 2023-02-18

**Authors:** Karen Chin Snyder, Weihua Mao, Joshua P. Kim, Justine Cunningham, Indrin J. Chetty, Salim M. Siddiqui, Parag Parikh, Jennifer Dolan

**Affiliations:** ^1^ Department of Radiation Oncology Henry Ford Health Detroit Michigan USA

**Keywords:** brain, inter‐fraction motion, MR coil, MRgRT

## Abstract

To evaluate the image quality, dosimetric properties, setup reproducibility, and planar cine motion detection of a high‐resolution brain coil and integrated stereotactic brain immobilization system that constitute a new brain treatment package (BTP) on a low‐field magnetic resonance imaging (MRI) linear accelerator (MR‐linac). Image quality of the high‐resolution brain coil was evaluated with the 17 cm diameter spherical phantom and the American College of Radiology (ACR) Large MRI Phantom. Patient imaging studies approved by the institutional review board (IRB) assisted in selecting image acquisition parameters. Radiographic and dosimetric evaluation of the high‐resolution brain coil and the associated immobilization devices was performed using dose calculations and ion chamber measurements. End‐to‐end testing was performed simulating a cranial lesion in a phantom. Inter‐fraction setup variability and motion detection tests were evaluated on four healthy volunteers. Inter‐fraction variability was assessed based on three repeat setups for each volunteer. Motion detection was evaluated using three‐plane (axial, coronal, and sagittal) MR‐cine imaging sessions, where volunteers were asked to perform a set of specific motions. The images were post‐processed and evaluated using an in‐house program. Contrast resolution of the high‐resolution brain coil is superior to the head/neck and torso coils. The BTP receiver coils have an average HU value of 525 HU. The most significant radiation attenuation (3.14%) of the BTP, occurs through the lateral portion of the overlay board where the high‐precision lateral‐profile mask clips attach to the overlay. The greatest inter‐fraction setup variability occurred in the pitch (average 1.08 degree) and translationally in the superior/inferior direction (average 4.88 mm). Three plane cine imaging with the BTP was able to detect large and small motions. Small voluntary motions, sub‐millimeter in magnitude (maximum 0.9 mm), from motion of external limbs were detected. Imaging tests, inter‐fraction setup variability, attenuation, and end‐to‐end measurements were quantified and performed for the BTP. Results demonstrate better contrast resolution and low contrast detectability that allows for better visualization of soft tissue anatomical changes relative to head/neck and torso coil systems.

## 1 INTRODUCTION

Magnetic resonance (MR)‐guided linear accelerators provide enhanced visualization of MR‐based soft tissue contrast relative to conventional x‐ray‐based methods, which allows for improved localization of radiotherapy treatments to soft tissue. Opportunities in MR guided radiotherapy (MRgRT) in the treatment of brain tumors is optimistic since MR imaging is the gold standard for visualization and detection of intracranial malignancies.^1^, ^2^ Furthermore, MR‐guided adaptive radiotherapy (MRgART) treatments allow for adjustment of the target volumes due to observed anatomical as well as functional changes on MR imaging throughout a radiotherapy treatment course.^3^, ^4^, ^5^

A limitation that has prevented the widespread adoption of intracranial MRgRT is the lack of MR‐linac specific head coils that seamlessly integrate with radiation therapy (RT) immobilization devices.^6^ Immobilization is necessary in RT to avoid inter‐ and intra‐fraction patient motion during treatment. Since MRgRT and MRgART require longer times on the machine, it is essential that the immobilization and imaging coil system be comfortable but rigid to minimize inter‐ and intra‐ fraction motion. Most diagnostic head coils cannot be utilized in RT setups since diagnostic head coils are radio‐opaque with narrow openings that prevent access to the head and space for immobilization devices.

Current MR‐linac head coils utilize a combination of body or head and neck coils supported on a bridge, or flat flexible surface coils embedded in radiolucent foam that are built around the RT mask system or placed directly on the patient.^7^, ^8^, ^9^ Placement of the anterior coil on top of the patient can be uncomfortable, impede breathing, and cause claustrophobia. Current methods used to improve head coils for intracranial MRgRT applications have focused on optimization of geometry, to increase proximity to the scan area, reproducibility, and novel coil arrays. Previous work has been performed to engineer holders for existing coils to improve proximity, integrate flexible coil arrays into moldable immobilization devices, design and optimization of flexible, light weight coils that can be placed on the patient or molded closely to the patient.^10^, ^11^, ^12^

The ViewRay MRIdian system (ViewRay, Cleveland, OH), a commercially available MR‐linac system, consists of a 0.35T split magnet MR scanner and a 6 MV flattening filter free linear accelerator.^13^ The original MRIdian intracranial imaging coil is a two‐piece head and neck surface coil placed posterior and anterior to the patient that is retrofitted with a CIVCO head immobilization system.^9^

The MRIdian A3i upgrade includes an optional brain treatment package (BTP) consisting of a high‐resolution brain coil and a designated Orfit immobilization system. The high‐resolution brain coil is a one‐piece coil system with six radio‐frequency (RF) receive channels. The brain coil design optimizes the geometry, allowing closer proximity to the patient anatomy, and utilizes a patent pending two turn coil loop,^14^ that work in concert to increase the signal to noise ratio (SNR) and improve image quality. In this study, we aim to evaluate the image quality, dosimetric properties, setup reproducibility of the immobilization, and motion detection ability of the MRIdian A3i BTP.

## 2 METHODS AND MATERIALS

### 2.1 ViewRay A3i brain treatment package

The BTP consists of a high‐resolution brain coil that was designed to fit smoothly over the MR‐safe cranial immobilization. The immobilization devices include two glass fiber Orfit HP PRO, MR‐Safe, Overlay Base Plates (Orfit, Wijnege, Belgium). The two identical base plates (apart from the table‐top indexing adaptors) are for use in CT simulation (CT overlay) and MR‐guided simulation/treatment (MRgRT overlay), respectively. In conjunction with the HP PRO base plate, a mold care pillow and Orfit thermoplastic SRS‐Fix Mask system is used to immobilize the patient. The Orfit SRS‐Fix Mask system is a 3‐point mask that uses high‐precision (HP) lateral, low profile clips that attach the mask to the base plate.

The MR high‐resolution brain coil consists of a 6‐channel RF receiver coil embedded in radiolucent foam covering. The brain coil stands independently, not resting on the patient, and the electronic circuitry is superior to the patient and not within the primary radiation beam. The brain coil slides over the overlay base plate, to circumvent the patient's head. The brain coil is not indexed to the board and can be moved relative to the board in the superior, inferior direction and laterally, Figure 2. The high‐resolution brain coil can be used to acquire true fast imaging with steady‐state free precession (TRUFI), T1 weighted and T2 weighted MR images in clinical mode. The TRUFI scans can be acquired with a minimum sub‐millimeter isotropic resolution of 0.75 mm, and T1 and T2 scans can be acquired at a minimum 1 mm isotropic resolution. Images can be acquired in one of three acquisition modes (fast, faster, fastest). The acquisition mode sets the parallel imaging technique used to accelerate the image acquisition.^15^ Other imaging sequences can be acquired on the Siemens Avanto, A Tim+Dot system, and MR console (Siemens Healthcare GmbH, Erlangen, Germany).

A CT simulation coil with neither electronic circuitry nor receiver coils embedded in the radiolucent foam is also included. During treatment simulation it is not necessary to scan the treatment setup with the CT simulation coil. Because the CT simulation coil does not have any of the electronic circuitry or RF coils, it does not add additional CT/electron density information. It can be used during simulation to ensure that the patient setup can fit comfortably within the brain coil.

### 2.2 Image evaluation

Image quality tests were performed using the high‐resolution brain coil. SNR and uniformity tests were performed in a Siemens 170 mm diameter spherical head phantom filled with a Copper solution (Siemens, Erlangen Germany). The SNR in the transverse, sagittal, and coronal planes were obtained using a 171 cm^2^ region of interest (ROI), acquired with pre‐scan normalization off and calculated using Equation (1).

(1) $$\mathrm{SNR}\;=\frac{\mathrm{ROI}\;\mathrm{Mean}\;\mathrm{Signal}\;\times \;0.66}{\mathrm{ROI}\;\mathrm{Noise}\;\mathrm{SD}}\;$$

Multi‐channel data for the head coil was also obtained by analyzing the transverse, uncombined images.

Uniformity was obtained with the same ROI as the SNR test but with pre‐scan normalization on. The percentage uniformity (U%) was calculated using Equation (2).

(2) $$\begin{array}{c} U\%\;=\;100\times \left(1-\frac{\left(\mathrm{ROI}\;\mathrm{Signal}\;\mathrm{Max}-\mathrm{ROI}\;\mathrm{Signal}\;\mathrm{Min}\right)}{\left(ROI\;Signal\;Max+ROI\;Signal\;Min\right)}\right) \end{array}$$

Image quality analysis was performed on the Large ACR MRI phantom (JM Specialty Parts, Chattanooga TN) following ACR guidelines for ACR T1 and T2 Series.^16^ This includes geometric accuracy, high‐contrast spatial resolution, slice thickness accuracy, slice position accuracy, image intensity uniformity, percent‐signal ghosting, and low‐contrast object detectability, and contrast resolution.

Contrast resolution was assessed using the Full Low Contrast Object Detectability test. Slices 8 through 11, acquired on the ACR phantom contain the pattern with 10 spokes, each spike consisting of three circles of varying contrast. For the full test, the number of spokes that have all three circles discernible are counted on each of the four slices and then added together for a cumulative score.

**FIGURE 1:**
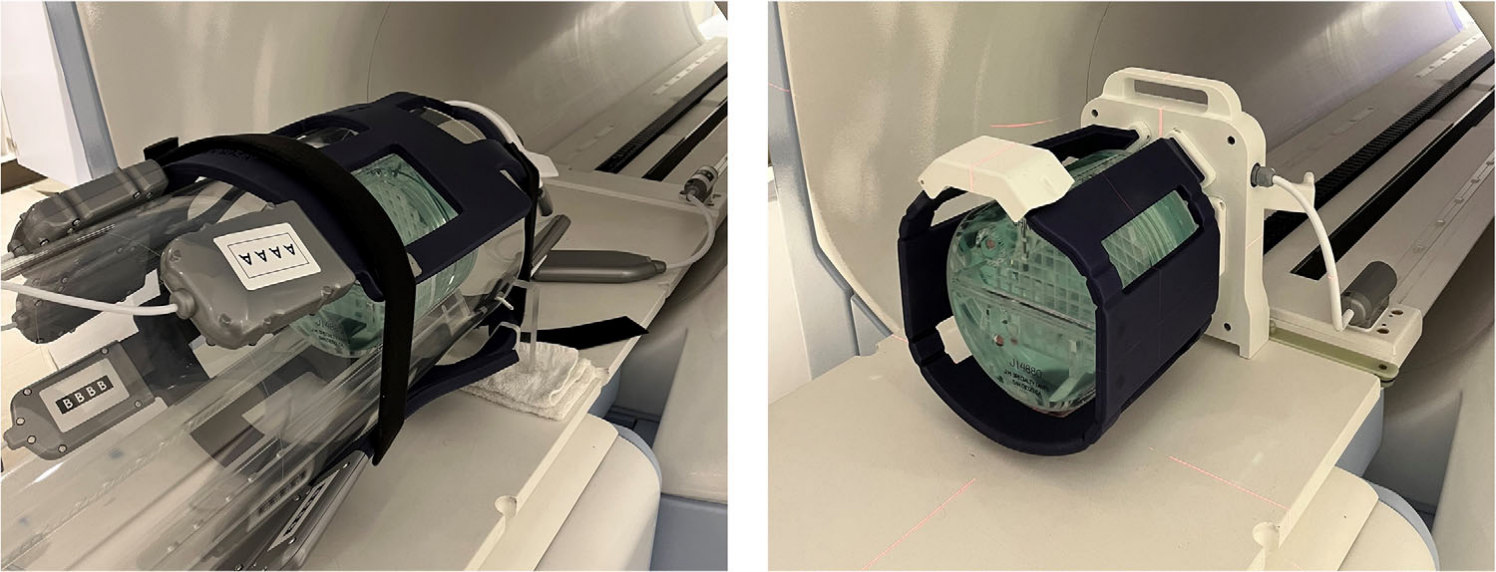
Large ACR phantom setup within the head and neck coil (left) and high‐resolution brain coil (right).

Image quality tests were repeated with the Torso coil, a two‐piece flexible coil system used for treatment of abdomen and thorax, as well as the Head and Neck coil, Figure 1. Image quality values for the three coil systems were compared.

### 2.3 Intracranial imaging

Four patients were imaged with the high‐resolution brain coil, under an IRB approved imaging study for low‐field MR. Three of the four patients were treated with the MRIdian A3i BTP. One patient was imaged, but not treated on the MRIdian.

TRUFI, T1, T2, and T2 Flair images were obtained as part of our clinical simulation protocol. All scans, excluding the T2 FLAIR, are available on the MRIdian console. The T2 FLAIR was acquired using the Siemens Avanto A Tim+Dot system. The radiotherapy treatment delivery system was disconnected, and an MRI was acquired in MR‐only mode on the Siemens MR platform. A TRUFI localization scan was acquired for planning and daily localization. An additional TRUFI with higher resolution than the localization scan, T1 and T2 scans were also acquired. Table 2 summarizes the scans acquired during patient simulation, scan parameters, and acquisition time. The scan times increase with decreasing slice thickness and increased field of view (FOV), Table 1. The values summarized in Table 2 took into consideration the time for scanning and resolution needed to achieve a clinically acceptable image.

**TABLE 1:** Image acquisition parameters for set field of view (FOV) 25 × 25 × 25 cm^3^.

		Isotropic voxel size (mm)			
Scan type	Acquisition mode	0.75	1.0	1.5	2.0
TRUFI	Fastest	4 min 25 s	3 min 2 s	1 min 9 s	29 s
	Faster	9 min 33 s	6 min 32 s	2 min 29 s	1 min 02 s
	Fast	17 min 9 s	11 min 43 s	4 min 20 s	1 min 35 s
T1	Faster	16 min 02 s	12 min 14 s	8 mm 53 s	6 min 54 s
T2	Faster	46 min 50 s	36 min 2 s	14 min 4 s	10 min 50 s

**TABLE 2:** Summary of MR scans acquired during patient MR simulation.

Scan description	Acquisition mode	Slice thickness	Scan FOV (x/y/z)	Scan time
TRUFI—localization	Fast	1.5 mm (isotropic)	20/25/24.7	2 min 35 s
TRUFI—high resolution	Fast	1 mm (isotropic)	21.8/25/24.4	7 min 22 s
T2	Faster	1.5 mm	20.7/24.8/20	14 min 4 s
T1 with contrast	Faster	1 mm	20/25/24.9	10 min 8 s
T2 FLAIR	NA	2.5 mm	20/25/17.5	8 min 52 sec

### 2.4 Radiographic and dosimetric evaluation of the integrated immobilization system

The Orfit CT and MRgRT overlays were scanned with a Philips Big Bore RT CT Scanner, (Amsterdam, Netherlands). Images were used to evaluate the dosimetric properties of the immobilization materials (scan parameters: 2 mm, 120 kVp, 450 mAs). The Hounsfield units (HU) were evaluated by individually contouring the inner and outer portions of the board and the ViewRay treatment couch adapter.

**FIGURE 2:**
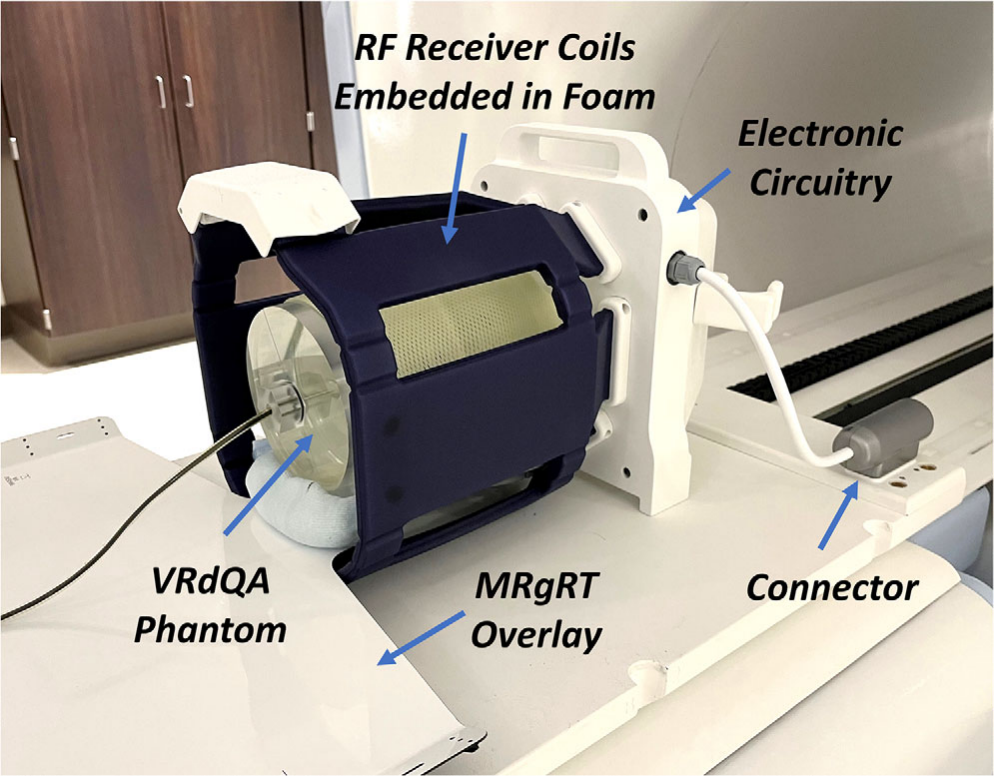
Setup for radiation attenuation measurements. The ViewRay Daily QA (VRdQA) phantom was immobilized in the Orfit mask on the Orfit MRgRT overlay integrated with the high‐resolution MR‐imaging coil. Please note the head coil is not indexed directly to the overlay board.

The ViewRay Daily QA (VRdQA) phantom was used in dosimetric testing by simulating the phantom in the Orfit SRS‐Fix Mask System. The VRdQA phantom is a cylindrical, water filled phantom (14 cm outer diameter, 20 cm in length), with an acrylic shell (6 mm thick) containing five cylindrical inserts that may be exchanged for different dosimetric inserts. An MR‐compatible ion chamber (Exradin A28MR from Standard Imaging, Middleton, WI) and corresponding insert were used for measurement in the VRdQA phantom. The VRdQA phantom was immobilized with a custom mold care pillow and Orfit SRS‐Fix mask. The phantom was arranged in the simulated treatment setup with the MR high‐resolution brain coil and immobilization device (Figure 2) and CT‐scanned using our institution head protocol (1 mm slice thickness, 120 kVp, 501 mAs). Photon attenuation properties of the MR high‐resolution brain coil were evaluated via HU acquired in the head protocol CT scan, Figure 6.

Dose was measured through the immobilization system and coils at 5 posterior‐oblique angles (Gantry 180, 160, 140, 120, 90). Calculated dose in the commercial Monte Carlo treatment planning system was compared to the measured dose using three different setups: the MRgRT overlay board (including HP lateral clips) without the high‐resolution brain coil (NC); the brain coil without the MRgRT overlay board (including HP lateral clips) (NO); and without either the MRgRT overlay or brain coil (NOC).

### 2.5 End‐to‐end testing

The VRdQA immobilized in the Orfit SRS‐fix Mask was utilized for dosimetric End‐to‐end testing. An MR simulation of the phantom was acquired using a TRUFI 1 × 1 × 1 mm^3^ isotropic scan with 25 cm FOV. A plan was created for a virtual brain PTV (180.72 cm^3^) to receive 50 Gy in 2 Gy fractions. The organs at risk included a virtual brainstem drawn in proximity to the PTV (but not overlapping) and surrounding normal “brain” tissue. The plan was created and delivered using a simple, MR‐primary workflow to simulate our conventionally fractionated brain treatment clinical workflow. A simple MR workflow begins with the rigid registration of a CT of the phantom setup to the MR simulation image. The CT values are then used in the background for electron density. Ten roughly equal spaced, step‐and‐shoot IMRT beams were utilized (Gantry angles: 354, 30, 66, 102, 138, 174, 210, 246, 282, 318) that simulate a typical beam arrangement for a partial brain treatment. Some gantry angles traverse through high‐attenuation areas of the ViewRay couch; however, those angles were not excluded in the study since they are modeled in the treatment planning system and often unavoidable in difficult cases. The plans were optimized using the “Objectives and Constraints” optimization algorithm and multileaf‐collimator leaves were sequenced with the maximum number of segments set to 60. The final dose was calculated with a 1 mm grid size, 1% uncertainty in dose prediction, with the magnetic field turned off as per our current clinical workflow.

### 2.6 Inter‐fraction setup evaluation

Custom Orfit SRS‐Fix Masks were created for four healthy volunteers. The volunteers were scanned three times to quantify inter‐fraction setup reproducibility and motion detection. From the three scans, 3 matches were performed (2 to 1, 3 to 1, and 3 to 2) to quantify the amount of motion between each scan. Scans were aligned to the two lateral ventricles, which are easily visualized in the TRUFI scans. Six degree of freedom matches with translation and rotational corrections were performed in the Eclipse treatment planning system (Varian, Palo Alto CA) using the mutual information registration algorithm with a ROI surrounding the ventricles.

### 2.7 Motion detection tests with multi‐planar tracking

In the MRIdian A3i platform, planar cine images can be acquired during treatment in a minimum of 1 or maximum of 3 orthogonal planes. Typically, in abdomen or thorax tracking, one sagittal image is used to track breathing motion. Three orthogonal planes can be used to better detect random motions such as roll and pitch from patient motion. The high‐resolution brain coil allows for tracking in all three planes. Due to the higher image resolution of the brain coil cine images (0.13 cm vs. 0.35 cm of the torso coil), the use of three orthogonal planes slows down the cine acquisition to 0.5 frames per second (fps), 0.9 fps for two orthogonal planes, or 1.8–2 fps for one orthogonal plane.

Treatment simulation of the four volunteers was performed in the ViewRay RealView, imaging only, workspace. The gantry is parked at gantry 0, to avoid any image artifacts during imaging. During treatment simulation, three orthogonal planes (axial, coronal, and sagittal) were acquired for the volunteers. The volunteers were instructed to perform a set of motions. The first motion was to wiggle their arms and legs. Next, they gently moved their head by looking to the left, right, up, and down. Finally, the head motions were repeated, but in a more forceful manner.

**FIGURE 3:**
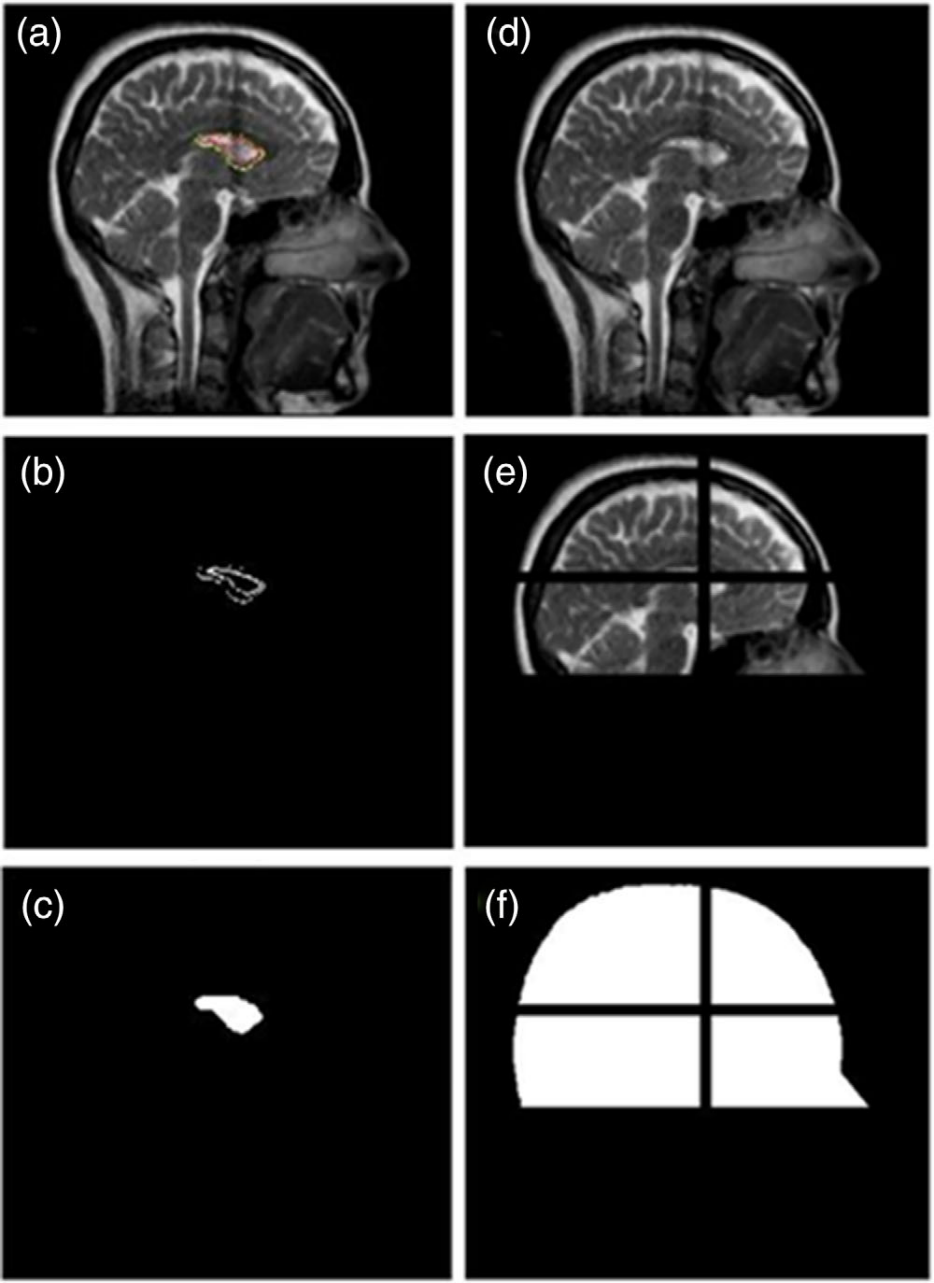
Image processing. (a) Cropped cine image; (b) target and tracking boundary were detected on the first frame of images; (c) contours filled; (d) cine image without contours; (e) detect artifacts due to fast orthogonal imaging; (f) region of interest for registration.

**FIGURE 4:**
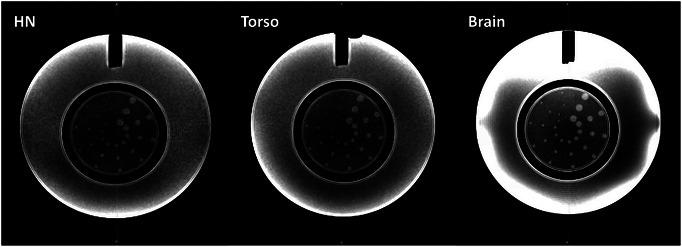
Slice 11 of ACR Large Phantom demonstrating the ten spokes of low‐contrast objects images acquired with the head and neck coil (left), torso coil (center) and high‐resolution brain coil (right). Window and level differ due to difference in intensity values between the images.

**FIGURE 5:**
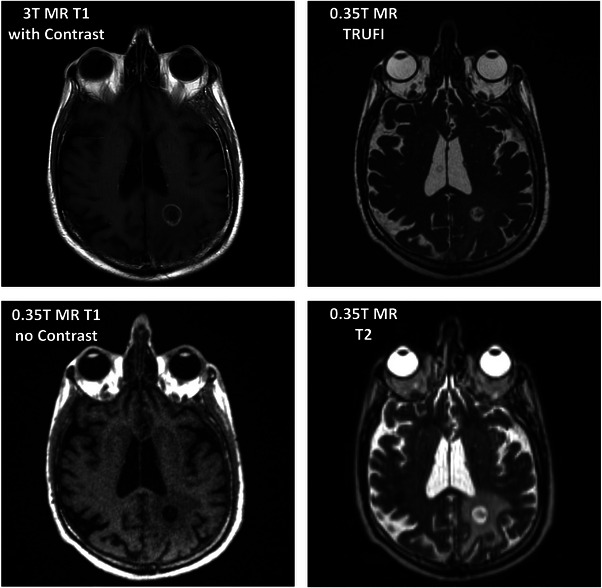
(a) Diagnostic axial T1 with contrast. (b) TRUFI, 1 mm isotropic, MRIdian A3i scan. (c) Axial T1, no contrast, 1 mm isotropic, MRIdian A3i scan. (d) Axial T2, 1.5 mm isotropic, MRIdian A3i scan.

**FIGURE 6:**
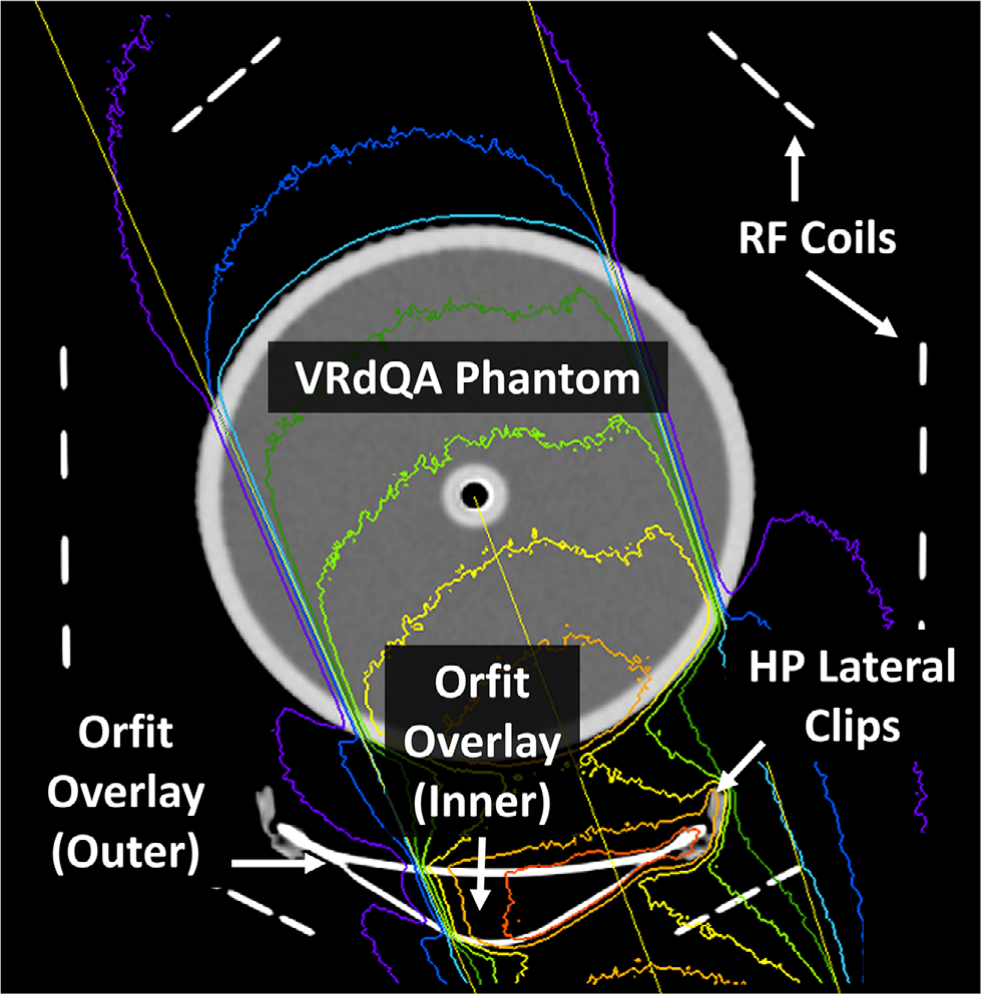
Axial view of electron density of high‐res imaging coils, Orfit overlay, lateral clips, and VRdQA phantom with posterior oblique beam, Gantry 160, 10 × 10 cm^2^ field size used in evaluation of attenuation.

The magnitude of the motion was evaluated in the three orthogonal views independently with an in‐house Matlab (MathWorks, Natic, MA) program, which is the supplementary material. Figure 3 demonstrates the image processing procedures: (1) loaded exported images as videos and cropped system added boundaries (Figure 3a); (2) detected contours (colored) for the target and tracking boundaries on gray scale images (Figure 3b); (3) filled the contours with internal Matlab functions (image dilate, fill, and erode) and used the center of the contour as the center of rotation (Figure 3c); (4) removed contours from cropped images by filling the contour pixels with average intensity of neighbor pixels (Figure 3d); (5) detected and removed the “crossline” artifacts due to fast orthogonal imaging in other views by comparing summations along the orthogonal direction (Figure 3e); (6) excluded the crossline artifacts and only included skull level in the ROI for image registration; and (7) registered subsequent image frames with the first frame with two‐dimensional shifts and one‐dimensional rotation. Pearson correlation coefficients in the ROI were used as similarity measurement (Figure 4).

## 3 RESULTS

### 3.1 Image quality

Table 3 summarizes the signal and noise ratio (SNR) and percent uniformity (U%) in three orthogonal planes for the torso, head and neck coil, and high‐resolution brain coil. Table 4 summarizes ACR tests performed in the large ACR phantom for the torso, head and neck coil, compared to the high‐resolution brain coil. Figure 4 demonstrates the difference in images between the two coils for the ACR Large MRI Phantom at slice 11, demonstrating the low‐contrast objects. The low‐contrast object detectability results are quantitatively equivalent between the torso, head and neck, and brain coils. In the full low contrast object detectability test, the high‐resolution brain coil shows an improvement by detecting 37 contrast features compared to 28 of the head and neck coil.

**TABLE 3:** Summary of normalized signal to noise ratio (SNR) and percent uniformity (U%) in three orthogonal planes in the transverse, sagittal and coronal planes.

	Head and neck coil		Torso coil		Brain coil	
Plane	SNR	U%	SNR	U%	SNR	U%
Transverse	54.40	85.09	51.58	88.84	46.03	86.78
Sagittal	48.92	86.80	45.62	88.31	36.30	77.31
Coronal	39.10	86.52	38.39	88.53	39.71	86.34

**TABLE 4:** Summary and comparison of ACR tests performed on large ACR phantom with head and neck, torso, and high‐resolution brain coil.

Test name	Evaluation method	Criteria	Head and neck coil	Torso coil	Brain coil
Abbreviated low‐contrast object detectability	Number of contrast feature, slice 11	Number of Spokes (≥7 for nominal field strength < 1.5T)	10	10	10
	Number of contrast feature, slice 10		9	9	10
	Number of contrast feature, slice 9		8	8	9
	Number of contrast feature, slice 8		1	3	8
Full low‐contrast object detectability	Number of contrast feature, sum of slices 8–11		28	30	37
Geometric accuracy	Measure length of ACR phantom	A‐P (19 cm)	19.03 cm	19.01 cm	18.95 cm
		L‐R (19 cm)	19.07 cm	19.03 cm	19.06 cm
		S‐I (14.8 cm)	14.83 cm	14.81 cm	14.75 cm
Spatial resolution	Slice 7, per ACR guidelines	A‐P	0.9 mm	0.9 mm	0.9 mm
		R‐L	0.9 mm	0.9 mm	0.9 mm
Uniformity	Slice 7, per ACR guidelines	Low	702.75	741.27	2193.23
		High	759.16	757.2	2517.25
		PIU (%)	96.1	98.9	93.1
Percent signal ghosting	Slice 7, per ACR guidelines	Top	20.6	24.7	26.4
		Bottom	21.2	23.3	26.1
		Left	33.5	36.9	24
		Right	40.9	35	21
		Large ROI	733.2	749.6	2295.3
		Ghosting ratio	0.022	0.016	0.002

### 3.2 Intracranial imaging

Figure 5 demonstrates four different MR sequences for the same patient at the level of the lesion. Figure 5a. demonstrates a diagnostic post‐contrast T1 weighted MR image, scanned on a diagnostic GE Signa HDxt 1.5T MR, with a 2.75 mm slice thickness. Compared to Figures 5b–d MR images obtained on the 0.35T ViewRay MR with the high‐resolution brain coil.

### 3.3 Attenuation of immobilization and MR imaging coil

Table 5 summarizes the HU values of the CT and MRgRT overlays, the couch adapter, and the RF receiver coils of the brain coil. It was noted that the HUs for the superior, outer portion of the Orfit overlay were greater than the inferior, outer portion specifically in the areas where the HP lateral clips attach to the overlay board.

**TABLE 5:** Evaluation of HU values for inferior and superior portions of Orfit overlay, couch adapter for the MRgRT overlay and the coils.

	Inferior Orfit overlay—outer	Inferior Orfit overlay—inner	Superior Orfit overlay—outer	Superior Orfit overlay—inner	Adapters	RF coils
MRgRT Overlay	−263 ± 253.7 (138)	−921 ± 13.6 (−808)	310 ± 242.5 (1136)	−922 ± 20.3 (−667)	305 ± 127 (1121)	572 ± 240 (869)
CT Overlay	−224 ± 265 (192)	−923 ± 18.8 (−834)	332 ± 281.5 (993)	−932 ± 10.2 (−882)	NA	

*Note*: Mean +/− standard deviation (maximum).

The greatest attenuation through the treatment setup, 3.14%, occurs at Gantry 160. The beam traverses through the RF coils and the portion of the overlay board that the mask attaches to with the HP lateral clips, as demonstrated in Figure 6. Table 6 summarizes the difference between measured and calculated dose with portions of the Orfit immobilization system excluded in the calculations. To evaluate whether the overlay, RF coils, or both can be excluded in dose calculation, the differences between calculated and measured were also evaluated. The results for the calculation without the coils were all within 2%, where the measurement uncertainty was about 1%.

**TABLE 6:** Comparison of measured and calculated dose with portion of the Orfit immobilization excluded in dose calculation.

		Calculated TPS dose				Percent difference (measured‐calculated)				
Gantry angle	Measured dose	ALL	NC	NO	NOC	ALL	NC	NO	NOC	TPS calculated (NOC—ALL)
180	71.3	71	70.7	72.8	72.9	0.43%	0.86%	−2.05%	−2.18%	2.68%
160	70.7	70	70.4	72.4	72.2	1.06%	0.49%	−2.29%	−2.02%	3.14%
140	69.0	69	70	69.6	69.5	0.04%	−1.39%	−0.82%	−0.68%	0.72%
120	74.2	73.4	72.6	73.2	72.9	1.12%	2.23%	1.40%	1.81%	−0.68%
90	83.9	83	83.4	83.4	82.9	1.15%	0.66%	0.66%	1.27%	−0.12%

Abbreviations: NC, no coils; NO, no overlay; NOC, no overlays or coils.

### 3.4 Dosimetric results of end‐to‐end

Table 7 summarizes the end‐to‐end point dose measurements and calculations for the cranial plan with different portions of the immobilization excluded from the dose calculation.

**TABLE 7:** Ion chamber, point dose measurement for cranial plan measured in VRdQA phantom with different immobilization included in dose calculation.

Plan	Measured dose	Calculated dose	Percent difference (%)
Plan including overlay and coils	206.06	206.8	−0.36
Plan with no coils, only overlay		208.3	−1.06
Plan with no overlay, only coils		209.2	−1.52
Plan with no overlay or coils		210.4	−2.1

### 3.5 Inter‐fraction variability and motion detection

Table 8 summarizes the maximum absolute change in magnitude from one image to another for all four healthy volunteers. The largest variability in setup occurred in the pitch (4.3 degrees) and correspondingly the superior/inferior direction, with an average setup error of 4.88 mm.

**TABLE 8:** Inter‐fraction setup variability.

	Rotation (degree)			Translation (mm)		
	Roll	Pitch	Yaw	LAT	AP/PA	Sup/Inf
Average	0.51	1.08	0.67	1.22	3.91	4.88
StDev	0.30	1.21	0.39	1.17	6.31	5.67
Range (Min, Max)	(0, 1.1)	(0.2, 4.3)	(0.1, 1.4)	(0.2, 4.1)	(0.1, 20.2)	(0.4, 18.8)

*Note*: Average, minimum, and maximum values for rotational and translational setup differences over four healthy volunteers.

**FIGURE 7:**
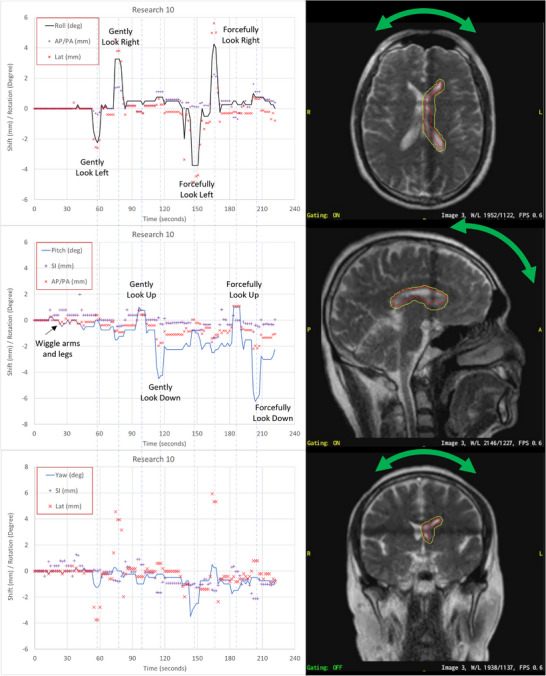
Tracked motion in axial, sagittal, and coronal views of one volunteer (left) and cine images showing corresponding views (right). Red contour is the tracking contour and the yellow contour is a 3 mm expansion used to gate the beam.

More than 400 image frames were acquired and analyzed for each of the four volunteers. Figure 7 illustrates the detected motions of one volunteer as function of time. The motions performed by the volunteer as directed were clearly detected. Small voluntary motions, sub‐millimeter in magnitude (maximum 0.9 mm), from motion of external limbs were detected. Table 9 lists the maximum motions detected for gentle and forceful movements.

**TABLE 9:** Maximum voluntary motion from volunteer scans.

	Gentle motion						Forceful motion					
	Rotation (degree)			Translation (mm)			Rotation (degree)			Translation (mm)		
Volunteer ID	Roll	Pitch	Yaw	Lat	AP/PA	SI	Roll	Pitch	Yaw	Lat	AP/PA	SI
#3	2.8	1.3	0.5	3.9	1.0	1.6	4.7	2.8	0.8	5.8	1.7	2.1
#8	1.7	1.3	1.0	2.2	1.1	1.1	4.7	6.2	2.0	6.3	5.8	2.0
#9	2.3	2.0	0.8	1.1	0.9	1.2	3.5	5.3	2.2	2.5	3.5	2.8
#10	3.2	4.5	1.3	4.6	2.0	2.0	4.3	6.2	3.5	6.9	2.2	3.5

## 4 DISCUSSION

The design of the new high‐resolution brain coil has better contrast resolution and higher SNR in the coronal plane, relative to its predecessors: the torso and head and neck coils. This allows for better visualization of small intracranial anatomical structures and normal tissue features allowing clinicians to better visualize soft tissue changes. The ability to easily acquire T1 and T2 weighted MR images through the treatment delivery console allows for ease in obtaining images periodically through the course of treatment that can be used to monitor treatment response. The image quality is proportional to the acquisition time, and thus care must be taken when determining what image sequences should be acquired to maximize information necessary for contouring and to minimize table time for the patient. ACR Large MRI Phantom image evaluation confirms similar image quality, in line with results from the torso and head and neck coils at commissioning and acceptance.

Radiographic evaluation of the imaging coils and immobilization devices demonstrated that the RF coils embedded in the radiolucent foam have a high HU value. The coils are not directly indexed to the overlay or treatment table; therefore, the actual location of the high‐density components will vary from day to day. To avoid further dosimetric uncertainty, we chose to exclude the coils in the dose calculation rather than to optimize high‐dose gradients around the coil element placement.

From attenuation measurements and calculations, the BTP (including the overlay, coils, and mask) attenuates about 2%–3% of the beam. AAPM Task Group 176 recommends that couch and immobilization structures be included in the dose calculation to account for the 1%–2% dose perturbation.^17^ The design of the of the HP low‐profile clips allows for minimal perturbation of the beam, except in the area where the mask attaches to the overlay board. It is pertinent when planning with posterior oblique beams to include the overlay to account for attenuation. However, including the overlay can often be difficult because the MR FOV is limited to 25 × 25 × 25 cm^3^. The current FOV limits the amount of information in the MR scan and depending on the size of the patient's head the MR FOV may only be able to include the cranium and not the immobilization device. If MR is used as the primary image (MR primary) the background electron density from the CT is also truncated at the MR FOV. This prevents the electron density of the overlay to be included. If the FOV does not include the immobilization device, in some areas this could result in a 3% overestimation in the delivered dose. This can be mitigated by using beams sparingly through the area where the mask attaches to the overlay, and in the future the manufacturer may create a treatment planning system model of the immobilization device.

In the end‐to‐end test, with a more clinical geometry using a 10 beam IMRT plan showed that the measured difference without coils included in calculation was less than compared to the difference without an overlay. A 2% difference in measured and calculated dose was observed when no immobilization or coils were included in the dose calculation. This can vary depending on the location of the target and the contribution of dose through the posterior portions of the immobilization device. Furthermore, since the coils are not indexed to the immobilization device, the amount of attenuation from the coils may be negligible owing to the variability in placement of the coils.

Discrepancies in inter‐fraction variability occurred most frequently in the superior/inferior direction and the pitch. Our results are comparable to Babic et al, who compared inter‐fraction setup variability with cone‐beam CT (CBCT) of five frameless stereotactic mask systems, including the Orfit mask system.^18^ During setup for each image, the volunteer was allowed to adjust their head to get comfortable in the mold care pillow. If the mold care pillow is formed too loosely around the neck, this results in variability in neck positioning and impacts the setup in the superior/inferior and anterior/posterior directions. Furthermore, a few of our volunteers had less pronounced nasal structures, which results in less ability to index the mask to the volunteer thus causing additional uncertainty when setting the patient up in a mask. Since MRI uses nonionizing radiation, it is reasonable to re‐set up the patient and adjust for larger discrepancies in setup due to the pitch, which cannot be adjusted with the couch. However, since the MRIdian system does not allow for six degrees of freedom match, manually adjusting the pitch may require some trial and error. The use of a sagittal cine may be useful in guiding alignment of the patient before final fixation of the mask.

In the motion detection study, healthy volunteers within the mask system were coached to perform translational and rotational motions. The volunteers’ motions were detected by the system and correlated with the timing of the given directives. Overall, pitch values drifted slowly over the duration of the session, whereas the roll and yaw values returned to within 1 mm of the baseline image. The greatest variability while using the Orfit system was observed in the pitch. Therefore, when using this mask system for longer, hypo‐fractionated treatments, a bite block may be beneficial for improving reproducibility and minimizing the drift in pitch.

A limitation in this study is that the volunteer motions are highly subject‐specific, making it difficult to compare motions even between volunteers. Therefore, comparison to other mask and coil systems is not practical when using this limited population size. Although the amount of motion was initially alarming, the magnitude was similar to the values reported in a study by Mandila et al.^8^ that used MR cine images to assess motion in brain patients using the CIVCO mask system. Even with the best intentions, patient motion is unpredictable and unavoidable. With the ability to constantly monitor the patient using non‐ionizing radiation, patient motion can be tracked, and active gating or intra‐fraction realignment can be performed. The study showed that small motions can be detected, however, the accuracy and magnitude of the motions were not evaluated. Future studies include evaluation of the accuracy and sensitivity of motion detection using planar cine imaging to eventually give recommendations for thresholds to actively gate the beam as in surface monitoring and infrared motion monitoring systems.^19^

The imaging improvements in the BTP result in increased confidence to better visualize and localize soft tissue to treat intracranial lesions. Furthermore, improvements in immobilization and tracking allow for increased patient comfort and monitoring. The BTP demonstrates excellent potential to overcome current limitations to allow for more widespread adoption of intracranial MRgRT. One limitation to the study is intra‐fraction motion of the immobilization system was not evaluated. Intra‐fraction motion is used to evaluate the rigidity of the immobilization system over the time of treatment, where studies have shown that head motion increases with time in the mask.^20^ Future uses of the system include fractionated stereotactic and adaptive RT. Given the longer treatment times, a thorough investigation of intra‐fraction motion should be conducted.

## 5 CONCLUSION

Imaging tests, inter‐fraction setup variability, attenuation, and end‐to‐end measurements were quantified for the BTP. Results demonstrate better contrast resolution and low contrast detectability that allows for better visualization of soft tissue anatomical changes relative to head/neck and torso coil systems.

## AUTHOR CONTRIBUTIONS

Karen Chin Snyder designed, directed the project, and wrote the manuscript. Jennifer Dolan and Justine Cunningham helped conceive the study. Joshua P. Kim and Weihua Mao performed data collection and analysis. All authors provided critical feedback and helped shape the research and final manuscript.

## CONFLICT OF INTEREST STATEMENT

Henry Ford Health holds a research agreement with ViewRay.

## Supporting information

SUPPORTING INFORMATION

## References

[1] Chin S , Eccles CL , Mcwilliam A , et al. Magnetic resonance‐guided radiation therapy: a review. J Med Imaging Radiat Oncol. 2020;64(1):163‐177. 31646742 10.1111/1754-9485.12968

[2] Kupelian P , Sonke J‐J . Magnetic resonance–guided adaptive radiotherapy: a solution to the future. Semin Radiat Oncol. 2014;24(3):227‐232. 24931098 10.1016/j.semradonc.2014.02.013

[3] Maziero D , Straza MW , Ford JC , et al. MR‐Guided radiotherapy for brain and spine tumors. Front Oncol. 2021;11:626100. 33763361 10.3389/fonc.2021.626100 PMC7982530

[4] Mehta S , Gajjar SR , Padgett KR , et al. Daily tracking of glioblastoma resection cavity, cerebral edema, and tumor volume with MRI‐guided radiation therapy. Cureus. 2018;10(3):e2346. 29796358 10.7759/cureus.2346 PMC5959724

[5] Cao Y , Tseng C‐L , Balter JM , Teng F , Parmar HA , Sahgal A . MR‐guided radiation therapy: transformative technology and its role in the central nervous system. Neuro‐oncol. 2017;19(suppl 2):ii16‐ii29. 28380637 10.1093/neuonc/nox006 PMC5463498

[6] Glide‐Hurst CK , Paulson ES , Mcgee K , et al. Task group 284 report: magnetic resonance imaging simulation in radiotherapy: considerations for clinical implementation, optimization, and quality assurance. Med Phys. 2021;48(7):e636‐e670. 33386620 10.1002/mp.14695 PMC8761371

[7] Cuccia F , Alongi F , Belka C , et al. Patient positioning and immobilization procedures for hybrid MR‐linac systems. Radiat Oncol. 2021;16(1):183. 34544481 10.1186/s13014-021-01910-6 PMC8454038

[8] Mandija S , D'agata F , Navest RJM , et al. Brain and head‐and‐neck MRI in immobilization mask: a practical solution for MR‐only radiotherapy. Front Oncol. 2019;9:647. 31380283 10.3389/fonc.2019.00647 PMC6650525

[9] Konnerth D , Eze C , Nierer L , et al. Novel modified patient immobilisation device with an integrated coil support system for MR‐guided online adaptive radiotherapy in the management of brain and head‐and‐neck tumours. Tech Innov Patient Support Radiat Oncol. 2021;20:35‐40. 34841095 10.1016/j.tipsro.2021.11.002 PMC8605429

[10] Mcgee KP , Campeau NG , Witte RJ , et al. Evaluation of a new, highly flexible radiofrequency coil for MR simulation of patients undergoing external beam radiation therapy. J Clin Med. 2022;11(20):5984. 36294304 10.3390/jcm11205984 PMC9604708

[11] Tyagi N , Zakian KL , Italiaander M , et al. Technical note: a custom‐designed flexible MR coil array for spine radiotherapy treatment planning. Med Phys. 2020;47(7):3143‐3152. 32304237 10.1002/mp.14184 PMC8353548

[12] Balter JM , Gupta D , Kim MM , et al. Clinical evaluation of a receiver coil custom designed for MR simulation of immobilized patients. MReadings: MR in RT. 7th ed. Journal of Applied Clinical Medical Physics. 2021;7:43‐47.

[13] Mutic S , Dempsey JF . The ViewRay system: magnetic resonance‐guided and controlled radiotherapy. Semin Radiat Oncol. 2014;24(3):196‐199. 24931092 10.1016/j.semradonc.2014.02.008

[14] Frass‐Kriegl R , Hosseinnezhadian S , Poirier‐Quinot M , Laistler E , Ginefri J‐C . Multi‐loop radio frequency coil elements for magnetic resonance imaging: theory, simulation, and experimental investigation. Front Phys. 2020;7:237.

[15] Deshmane A , Gulani V , Griswold MA , Seiberlich N . Parallel MR imaging. J Magn Reson Imaging. 2012;36(1):55‐72. 22696125 10.1002/jmri.23639 PMC4459721

[16] Radiology, A.C.o . Phantom Test Guidance for Use of the Large MRI Phantom for the MRI Accreditation Program . 2018. 4/17/18 [cited 2022 8/1/2022]. https://www.acraccreditation.org/‐/media/ACRAccreditation/Documents/MRI/LargePhantomGuidance.pdf?la=en

[17] Olch AJ , Gerig L , Li H , Mihaylov I , Morgan A . Dosimetric effects caused by couch tops and immobilization devices: report of AAPM Task Group 176. Med Phys. 2014;41(6Part1):061501. 24877795 10.1118/1.4876299

[18] Babic S , Lee Y , Ruschin M , et al. To frame or not to frame? Cone‐beam CT‐based analysis of head immobilization devices specific to linac‐based stereotactic radiosurgery and radiotherapy. J Appl Clin Med Phys. 2018;19(2):111‐120. 29363282 10.1002/acm2.12251 PMC5849846

[19] Wang H , Xu Z , Grantham K , et al. Performance assessment of two motion management systems for frameless stereotactic radiosurgery. Strahlenther Onkol. 2021;197(2):150‐157. 33047151 10.1007/s00066-020-01688-8 PMC7840652

[20] Mangesius J , Seppi T , Weigel R , et al. Intrafractional 6D head movement increases with time of mask fixation during stereotactic intracranial RT‐sessions. Radiat Oncol. 2019;14(1):231. 31852497 10.1186/s13014-019-1425-7 PMC6921566

